# Predicting Positive and Negative Relationships in Large Social Networks

**DOI:** 10.1371/journal.pone.0129530

**Published:** 2015-06-15

**Authors:** Guan-Nan Wang, Hui Gao, Lian Chen, Dennis N. A. Mensah, Yan Fu

**Affiliations:** Web Sciences Center, School of Computer Science and Engineering, University of Electronic Science and Technology of China, Chengdu, China; Hangzhou Normal University, CHINA

## Abstract

In a social network, users hold and express positive and negative attitudes (e.g. support/opposition) towards other users. Those attitudes exhibit some kind of binary relationships among the users, which play an important role in social network analysis. However, some of those binary relationships are likely to be latent as the scale of social network increases. The essence of predicting latent binary relationships have recently began to draw researchers' attention. In this paper, we propose a machine learning algorithm for predicting positive and negative relationships in social networks inspired by structural balance theory and social status theory. More specifically, we show that when two users in the network have fewer common neighbors, the prediction accuracy of the relationship between them deteriorates. Accordingly, in the training phase, we propose a segment-based training framework to divide the training data into two subsets according to the number of common neighbors between users, and build a prediction model for each subset based on support vector machine (SVM). Moreover, to deal with large-scale social network data, we employ a sampling strategy that selects small amount of training data while maintaining high accuracy of prediction. We compare our algorithm with traditional algorithms and adaptive boosting of them. Experimental results of typical data sets show that our algorithm can deal with large social networks and consistently outperforms other methods.

## Introduction

Social network sites (SNSs) have grown steadily over the course of technological innovation. The social network perspective which focuses on relationships among people (or organizations or other social entities) is increasingly attracting the attention of academic and industry researchers [1]. In online social networks such as Epinions and Slashdot, users often give ratings to items or users, and tag other users as "friends" or "foes" [2]. From graph theory perspective, a directed link between two nodes (i.e., users) is assigned a positive or a negative sign, according to the initiator's positive (e.g., trust, support, or endorse) or negative (e.g., distrust, opposition, or dispute) attitude toward the other user, respectively. Those positive or negative attitudes exhibit the binary relationships among users, which can be used to capture the basic characteristics and the structure of the social network [2, 3], understand the propagation of trust and distrust in the social network [4, 5], recommend new friends to users in the social network [6–10], and etc.

Though relationship analysis plays an important role in the study of social networks, some relationships are more likely to be latent in large-scale social networks [11, 12]. Link prediction [9, 13] is the most fundamental method used to estimate the existence of links or attributes of links between two nodes, relying on the available information in the observed network. Generally, there are three main strategies for link prediction. The first strategy is to investigate node similarity in an unsupervised fashion [14–16]. The basic idea of this strategy is to assign a similarity score to each pair of nodes, and a link is expected to have higher likelihood of connecting a pair of nodes with higher similarity score. The second strategy is to consider both the structure of network and node attributes for machine learning, and treat link prediction as a binary classification problem [8, 17–19]. The third strategy is to predict links based on the underlying structures abstracted from observed networks using probabilistic models, such as hierarchical structure models [20], latent space models [21], and stochastic relational models [22].

Traditional link prediction often focuses on the likelihood of the existence of a link between two nodes in an unweighted and undirected network. However, link prediction should also be extended to take into account the directions and weights of links. Recently, predicting links with binary (i.e., positive and negative) relationships have attracted a considerable amount of attention [12, 22, 23]. There are two different theories commonly used for positive and negative relationships prediction: structural balance theory [24, 25] and social status theory [22, 26]. Structural balance theory originated in social psychology in the mid-20th-century. The main idea of this theory is to consider the possible patterns in which triadic relations of three individuals can be constructed, and points out that balanced triads (e.g., two friends with a common enemy or friend) are more plausible than unbalanced triads (e.g., two enemies with a common friend or enemy) in real networks. Social status theory is based on the directed network. This theory posits that each directed link with a positive/negative relationship denotes that the target node has a higher/lower status than the source node. By observing real social networks, Guha et al [22] succeeded in predicting the unknown trust/distrust relationships based on social status theory. Leskovec et al [23] proposed a model to predict the positive and negative relationships based on the aforementioned theories. Although Leskovec’s model achieved higher prediction accuracy than the former algorithm [22], it used logistic regression which only considered simple dependencies among the variables, and its hypothesis space may be too small to properly represent the data. Ye et al [12] focused on how to reliably and effectively infer the relationships in a newly formed social network from a mature network. However, calculating indicators (e.g., betweenness centrality) in a large-scale network structure will take too much time and space.

To deal with large-scale social network, in this paper we propose a machine learning algorithm called ESS (Extracted Segment-based SVM) for predicting positive and negative relationships. To ensure high prediction accuracy for links of low *embeddedness* [1] (the number of common neighbors of two endpoints of an edge), in the algorithm we propose a segment-based training framework to split the training set into two parts according to their embeddedness, and build two more precise classifiers on the two sub-training set, respectively. Considering the restricted memory space and running time for training, we employ an effective data sampling method to reduce the size of training data set as well as maintaining high prediction accuracy.

## Materials and Methods

**Fig 1:**
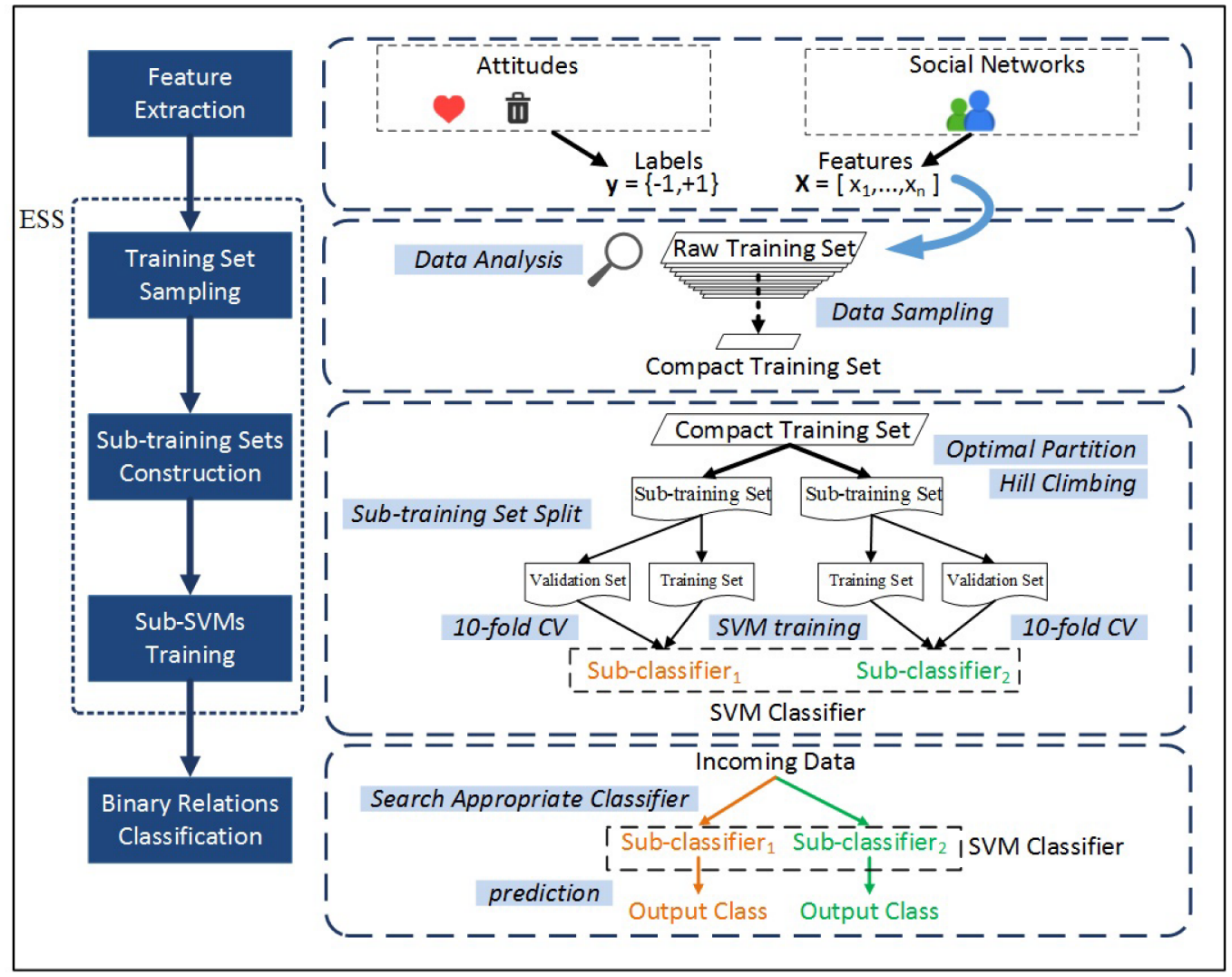
An overview of our algorithm. Our algorithm extracts features of edges from social network based on structural balance theory and status theory, and constructs a compact training set from the raw data by sampling strategies. While training two sub-SVMs on the split sub-training sets, our algorithm searches the optimal split of training set through maximizing the 10-fold cross-validation accuracy by using Hill Climbing method. After training sub-SVMs, predictions can be done by assigning the incoming data to the appropriate sub-SVM based on *EM*. (drawn by GNW)

We predicted positive and negative relationships on two large social networks from Epinions [27] and Slashdot [28] (S1 Datasets). Epinions is a site for product reviews, where users can decide whether to ''trust'' each other or not. Slashdot is a site that provides technology related news; it allows users to tag each other as friends or foes. We scrutinized the two networks, and also counted reciprocal links (two users hold the same attitude to each other). Data description and basic statistical properties are listed in Table 1. These two data sets are suitable for our experiments as links are explicitly labeled as positive or negative in those networks. Once the training set is obtained, our model (ESS) which can continuously self-improve will be trained on it. The objective of ESS is to learn a classification function from training set for predicting the latent labels (i.e. positive or negative relationships) in the network. Fig 1 presents the overview of ESS.

**Table 1:** Statistics of datasets from Epinions and Slashdot.

Characteristics	Epinions	Slashdot
**Edges**	841,372	549,202
**Nodes**	131,828	82,144
**Positive Edges**	85.3%	77.4%
**Negative Edges**	14.7%	22.6%
**Reciprocal Edges**	30.9%	17.7%

### Preliminary

**Fig 2:**
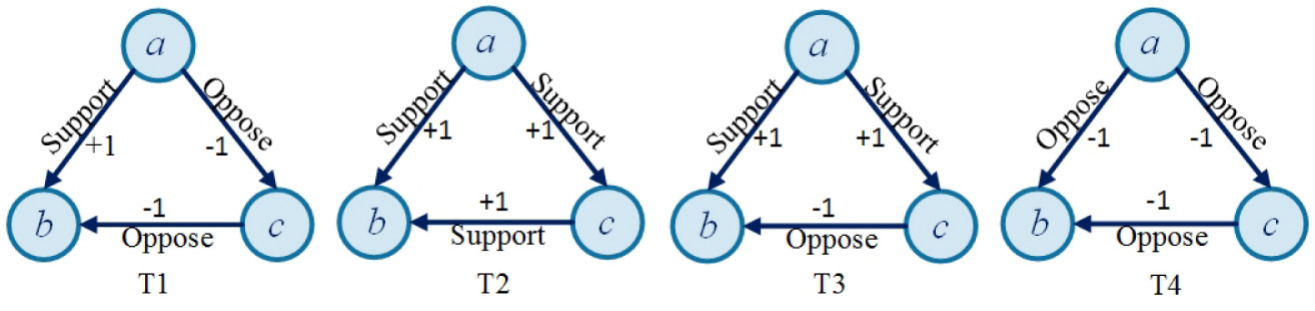
Examples of positive and negative relationships consisting of user a, b, c. 'Support' belongs to the positive attitudes, while 'Oppose' belongs to the negative attitudes.

Members and their relationships in a social network can be formalized as a directed graph *G*(*V*, *E*, *R*), where *V* is a set of nodes representing users in the social network, *E*⊆*V* × *V* is a set of directed edges representing relationships between users, and *R* is a set of signs associated to *E*. On each edge *e*_*i*_∈*E*, we have a sign *r*_*ab*_∈*R* reflecting the attitude held by its start point *a* towards its end point *b*. *r*_*ab*_ equals 1 when *a* holds positive attitude towards *b*, and -1 if otherwise. Fig 2 illustrates some cases of relationships. If user *a* holds supportive attitude towards user *b*, then *r*_*ab*_ in the graph will be labeled as +1. In this way, the problem of positive and negative relationships prediction is formalized into predicting the hidden signs on edges in the directed graph.

**Fig 3:**
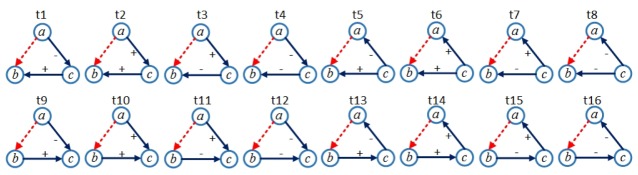
16 types of triangles determined by three nodes and a fixed unsigned edge (dotted).

Structural balance theory is concerning with balance and imbalance of sentiment relation in triadic relations. According to structural balance theory, we can categorize those four examples in Fig 2 into two kinds of structures. T1 and T2 are the balanced structures, namely, “my enemy's enemy is my friend", and "my friend's friend is my friend". T3 and T4 are unbalanced structures, namely, "my friend's enemy is my friend", and "my enemy's enemy is my enemy". Given a fixed unsigned edge (*a*, *b*), and the other two edges (*b*, *c*) and (*a*, *c*), it allows forming 16 types of triangles, which are illustrated in Fig 3. These types can also form complex structures. For instance, a combination of t10 and t14 stands for a triadic structure where *a* and *c* holds positive attitudes towards each other (reciprocal edge) while *b* holds positive attitude towards *c*.

Social status theory considers signed link formation based on an implicit ordering of the nodes. If user *a* thinks *b* has higher status than her, *a* will connect to *b* with a positive attitude (*r*_*ab*_ = 1), otherwise *a* will connect to *b* with a negative attitude (*r*_*ab*_ = -1). And social status is transitive: *r*_*ac*_ = 1 can be derived from the premises *r*_*ab*_ = 1 and *r*_*bc*_ = 1. The hidden signs on dotted edges of t2, t4, t14, t16 can be inferred according to the transitivity of status. Leskovec et al [23] defined an estimate of a node’s status. If node *n* has more incoming positive edges and outgoing negative edges, the value of status of *n* will become higher, and vice versa. The relationship on the edge is positive if it is directed from low status node to high status node, negative if otherwise. As social status theory is concerning endpoints of an edge and, therefore, not constricted to triadic structures, it is able to predict positive and negative relationships in some transitive structures which structural balance theory cannot handle.

### Feature extraction

An edge *e*_*i*_∈*E* can be treated as a feature vector **X**_*i*_ in the input space, and the sign on *e*_*i*_ can be treated as a label *y*_*i*_. An edge in the network has a series of features. We select features based on structural balance theory and social status theory. Suppose we have an edge *e*_*i*_ which points from node *a* to node *b* in *G*. Based on structural balance theory, *e*_*i*_ will participate in 16 types of triangles, as shown in Fig 3. $T_{i}=(T_{i}^{1},\ldots ,T_{i}^{16})$ is a vector consisting of the numbers associated with the 16 types of triangles that *e*_*i*_ is involved in. Based on social status theory, the sign of relationship on *e*_*i*_ is determined by the status of its endpoints. More precisely, we use the number of outgoing positive/negative edges from node *a*, and the number of incoming positive/negative edges to node *b*. $D_{i}=(d_{out}^{+}(a),d_{out}^{-}(a),d_{in}^{+}(b),d_{in}^{-}(b))$ is a vector consisting of the above four variables associated with *e*_*i*_. A long line of research in sociology has argued that if two individuals are connected by an embedded edge (an edge with common neighbors of its two endpoints), then this makes it easier for them to trust one another, and to have confidence in the integrity of the transactions (social, economic, or otherwise) that take place between them [29–32]. Consequently, we consider *embeddedness*, i.e. number of common neighbors of node *a* and node *b*, *EM*_*i*_ for short, as another feature of edge *e*_*i*_. We combine the variables {**T**_*i*_, **D**_*i*_, *EM*_*i*_} into **X**_*i*_ to describe an edge *e*_*i*_, that is **X**_*i*_ = (**T**_*i*_, **D**_*i*_, *EM*_*i*_).

### Training set sampling

Sampling of some massive data becomes important when collecting all of it or analyzing all of it is unreasonable. Suppose the training set contains *m* directed edges with explicit sign of relationships. For large network, if *m* is very big, the computational consumption will be relatively high, as standard SVM uses *O*(*m* ^3^) time and *O*(*m* ^2^) space for training. We need to reduce the size of the training set to *m'* ≪ *m* while maintaining valid training data. We proposed three simple sampling strategies: (i) *Random*. We randomly picked *m'* instances from the original training data to constitute the compact training set. (ii) *K-means cluster*. We first partitioned the original training data *S* into *K* cluster (used Euclidean Distance as the distance measure) [33], and then for each cluster *C*_*k*_, we randomly selected $\left\lfloor m\prime \left|C_{k}\right|/\left|S\right|\right\rfloor$ instances to constitute the compact training set. (iii) *Smallest out-degree*. Users that seldom express their opinions may be more cautious when showing their attitudes in the social network. Applying these instances as training data will exclude noise (e.g. some users may provide wrong attitudes intentionally or unintentionally). Therefore, we picked *m'* outgoing edges from the lowest out-degree user nodes.

### Sub-training sets construction

Since the triangle features $T_{i}=(T_{i}^{1},\ldots ,T_{i}^{16})$ of edge *i* are relevant only when endpoints *a* and *b* have neighbors in common, it is natural to expect that those features will be more effective with edges of greater *EM*. As Table 2 shows, Leskovec’s model will achieve higher prediction accuracy when classifying instances of higher *EM*. Figure A in S1 File shows that instances with high *EM* will contain more information about relationship which may lead to more accurate prediction. In other words, a single classifier may achieve low prediction accuracy when classifying instances of low *EM*. It is more appropriate to train sub-classifiers with instances of low *EM* and high *EM*, respectively. In order to achieve higher prediction accuracy, we designed a segment-based training framework, which trains sub-classifiers with instances of low *EM* and high *EM*, respectively. In fact, we can further construct sub-sub-classifiers by dividing the current sub-classifiers iteratively. The segment-based training framework uses Hill Climbing algorithm to pick a segmentation point *d* of training set *D*, which splits training set *D* into subsets *D*_1_ (0≤*EM*<*d*) and *D*_2_ (*d*≤*EM*). Figure B in S1 File shows the pseudo-code describing Hill Climbing process in our model. The goal of Hill Climbing is to ensure the overall cross-validation accuracy of the classifier *C*_1_ trained on *D*_1_ and the classifier *C*_2_ trained on *D*_2_ is the highest. To avoid overfitting of the sub-classifiers, we used 10-fold cross-validation [34] to "test" the sub-classifiers in the training phase. In 10-fold cross-validation, we randomly partitioned each sub-training set into 10 equal size subsamples. Of the 10 subsamples, a single subsample is retained as the validation data for testing the model, and the remaining 9 subsamples are used as real training data. The cross-validation process is then repeated 10 times (folds), with each of the 10 subsamples used exactly once as the validation data. The 10 results from the folds can then be averaged to produce a single estimation. The advantage of this method over repeated random sub-sampling is that all observations are used for both training and validation, and each observation is used for validation exactly once. The overall cross-validation accuracy over training set *D* is defined as follows:

$$p=\frac{\left|D_{1}\right|}{\left|D\right|}\cdot p_{C_{1}}+\frac{\left|D_{2}\right|}{\left|D\right|}\cdot p_{C_{2}}$$ (1)

where $p_{C_{i}}$ denotes the cross-validation accuracy of sub-classifier trained on sub-training set *D*_*i*_. Hill Climbing here tries to maximize *p* in Eq (1) by iteratively comparing solutions (i.e. different splits of *D*): it adopts the current best solution and continues to choose new solutions closest to the current best solution for comparison (i.e. move further up the hill). This iteration terminates when there are no better solutions on either side of the current solution (i.e. it has reached the peak). Figure C in S1 File shows prediction accuracy achieved by sub-classifiers with different segmentation points *d*. It is obvious that sub-classifiers will achieve higher prediction accuracy than that of a single one (the red point). Also, there is a peak in the curve, which can be quickly found by Hill Climbing. The use of segment-based training framework will make our model more robust than using a single classifier whose predictive power is likely to vary a great deal among instances of different *EM*s.

**Table 2:** Distribution of EM and prediction accuracy with different EMs.

	0≤*EM*<5	5≤*EM*<10	10≤*EM*<25	*EM*≥25
**Percentage of edges in Epinions**	43.78%	13.04%	17.69%	25.49%
**Prediction accuracy in Epinions**	90.53%	96.48%	96.51%	96.94%
**Percentage of edges in Slashdot**	80.90%	6.49%	6.48%	6.13%
**Prediction accuracy in Slashdot**	87.22%	88.75%	89.44%	95.36%

### Sub-SVMs training

SVMs can efficiently perform a non-linear classification using what is called the kernel trick, mapping their inputs into high-dimensional spaces. For a detailed introduction to the subject, please refer to support-vector networks [35]. SVM seeks to separate the data set into two classes using the optimal separating hyperplane (OSH) in the higher-dimensional space.

Given the training set {(**X**_*i*_, *Y*_*i*_), *i* = 1,…, *m*}, **X**_*i*_ is the *i*th input features, and *y*_*i*_∈{-1, 1} is a known binary target. In our case, **X**_*i*_ consists of the 21 features of an edge which were mentioned before. *y*_*i*_ indicates the positive/negative relationship on an edge. *y*_*i*_ is +1 for positive relationship on the edge and -1 for negative relationship. Points on either side of a separating hyperplane **W·X**+*b* = 0 have distances to that hyperplane. The smallest distance is called the margin of separation. The hyperplane is OSH when the margin is maximized. We can find OSH by solving the optimization problem:

$$\left\{ \begin{array}{l} \underset{w,b,\xi }{\text{min}}\;\frac{1}{2}w^{T}w+C\sum_{i}^{m}\xi_{i}\;\\ \begin{array}{c} subject\;to\;y_{i}\left(w^{T}x_{i}+b\right)\geq 1-\xi_{i},\;\\ \;\xi_{i}\geq 0,i=1,\ldots ,m. \end{array} \end{array}\right.$$ (2)

where *ξ*_*i*_≥0 is a slack variable introduced to generalize the problem to the non-separable case. *C* is a positive constant parameter used to control the trade-off between the training error and the margin. If we denote the *m* non-negative Lagrange multipliers as *α*_1_,…, *α*_*m*_ associated with *m* constraints of Eq (2), we can solve the optimization problem by SMO algorithm [36, 37]. Once the OSH is found from the training data, we can use it to predict the class a new instance belongs to, by simply checking on which side of the hyperplane it falls.

Our algorithm ESS trains two sub-SVMs on two sub-training sets, respectively. Incoming instances will be classified by the following piecewise classification function is:

$$y(x)=\{\begin{array}{c} \text{sgn}(\sum_{i}\alpha_{i}y_{i}K(x_{i},x)+b_{1})\text{ if value of }EM<d\\ \text{sgn}(\sum_{i}{\alpha^{\prime }}_{i}y_{i}K(x_{i},x)+b_{2})\text{ if value of }EM\geq d \end{array}\;$$ (3)

where *K*(**X**_*i*_, **X**) is the kernel function which mapped the input vector **X** to a higher-dimensional feature space where classes are linearly separable, and *d* is the segmentation point found by Hill Climbing. In this paper, we use a common kernel function i.e. RBF kernel $K(x_{i},x)=\text{exp}(-{\left\|x_{i}-x\right\|}_{2}^{2}/(2\sigma^{2}))$. There are two free parameters for a SVM model with RBF kernel: *C* (in Eq (2)) and *g* (i.e. 1/*σ* ^2^). The best parameter (*C* or *g*) is often unknown beforehand for a given problem [34], therefore we used grid-search algorithm [38] to identify good (*C*, *g*), so that the trained SVM can accurately predict unknown instances. In order to avoid overfitting and to improve generalization performance of the trained SVM, we used 10-fold cross-validation [34] to "test" SVM in the training phase. Given an edge in the social network, we input its feature vector into the piecewise classification function, and will get the label for the relationship on it.

## Results and Discussion

**Fig 4:**
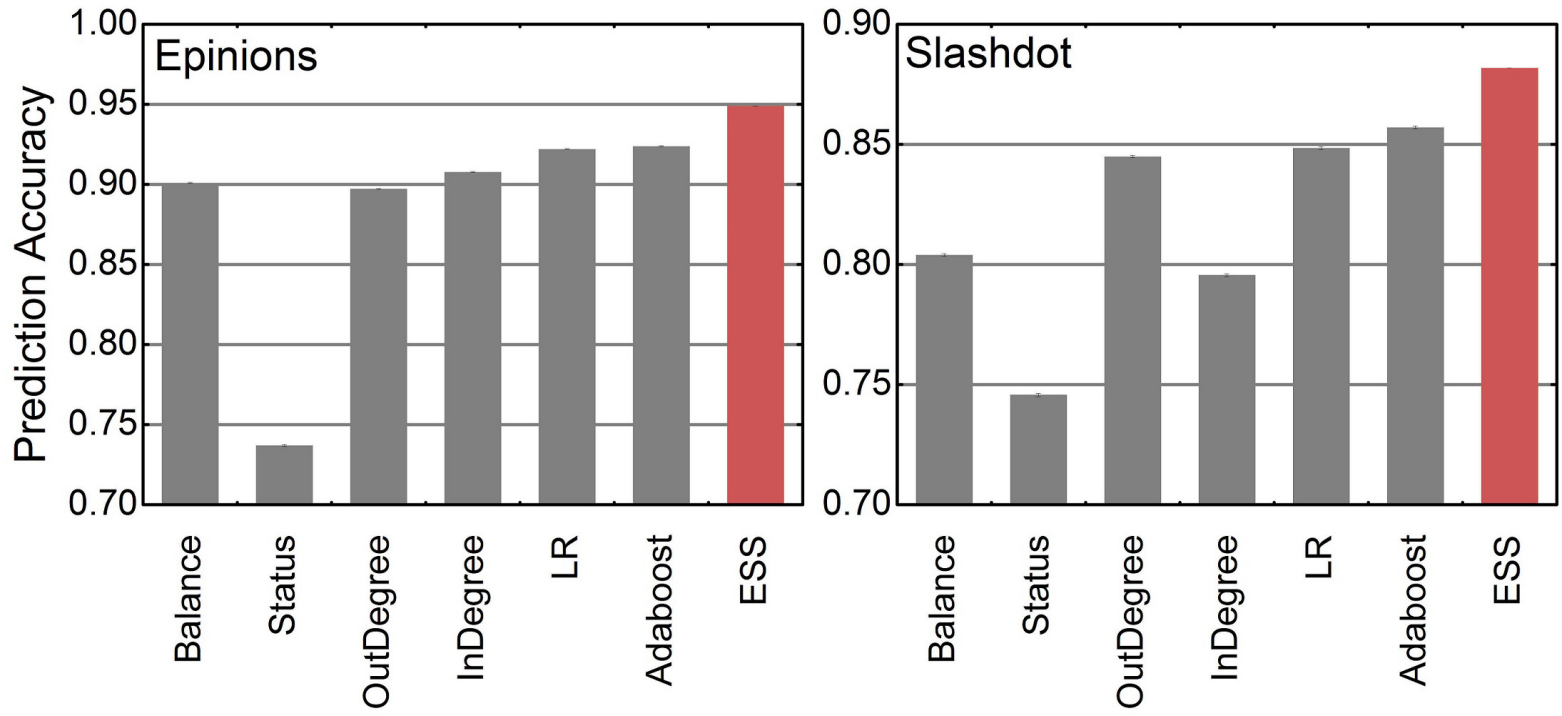
Prediction accuracy of 7 algorithms on Slashdot and Epinions. ESS is trained on a compact training set (3000 instances, i.e. the ratio of the size of the sampling size to the test set is around 0.01:1) extracted by sampling strategy (K-means cluster, *K* = 400) from the raw training set. Experiments are conducted on a PC with Intel Core i3-3220 CPU @ 3.30 GHz, 8 GB of RAM and 1.5 TB of hard disk.

We compared ESS with state-of-the-art algorithms [23], including heuristic algorithms and a supervised learning algorithm (LR) designed by Leskovec et al based on logistic regression. The heuristic algorithms include structural balance heuristic (Balance), social status heuristic (Status), out-degree heuristic (OutDegree) and in-degree heuristic (InDegree). LR uses a logistic regression classifier to combine a range of structural features into an edge sign prediction. Its structural features are extracted based on structural balance theory and social status theory. Balance chooses the sign for edge (*u*, *v*) that causes it to participate in a greater number of triads that are consistent with structural balance theory. Status defines an estimate of a node *x*’s status to be $\sigma (x)=d_{in}^{+}(x)+d_{out}^{-}(x)-d_{out}^{+}(x)-d_{in}^{-}(x)$. This gives *x*’s status benefits for each positive link it receives and each negative link it generates, and *x*’s status detriments for each negative link it receives and each positive link it generates. Status predicts a positive sign for (*u*, *v*) if *σ*(*u*) ≤ *σ*(*v*), and a negative sign otherwise. OutDegree predicts the majority sign based on the signs given by the edge initiator *u*. That is, It predicts positive sign if $d_{out}^{+}(u)\geq d_{out}^{-}(u)$. InDegree predicts the majority sign based on the signs received by the edge target *v*. That is, it predicts positive sign if $d_{in}^{+}(v)\geq d_{in}^{-}(v)$. In addition, we used AdaBoost algorithm [39] to integrate those five algorithms into a relatively strong classifier. AdaBoost creates the strong classifier by iteratively adding a weak learner in a “greedy fashion” i.e., it always chooses the weak learner with the lowest prediction error. During each round of training, a weak learner is added to the ensemble and weights of instances are adjusted to focus on the misclassified instances in previous rounds. We test the above algorithms on Slashdot and Epinions datasets, and use a 90/10 split for training and testing. Fig 4 shows the comparison of the prediction accuracy of the existing algorithms with ESS. On the average, ESS achieves prediction accuracy of 95.02% on Epinions and prediction accuracy of 88.19% on Slashdot. We also compared the area under the ROC curve (AUC) of our method with Leskovec’s model and Adaboost. The results shown in Table 3 indicate that our algorithm (ESS) outperforms other algorithms, even compared with the strong classifier constructed by AdaBoost.

**Table 3:** AUC for 3 algorithms on Slashdot and Epinions.

	Epinions	Slashdot
**ESS**	0.9759	0.9153
**Adaboost**	0.9648	0.9067
**LR**	0.9554	0.8998

**Fig 5:**
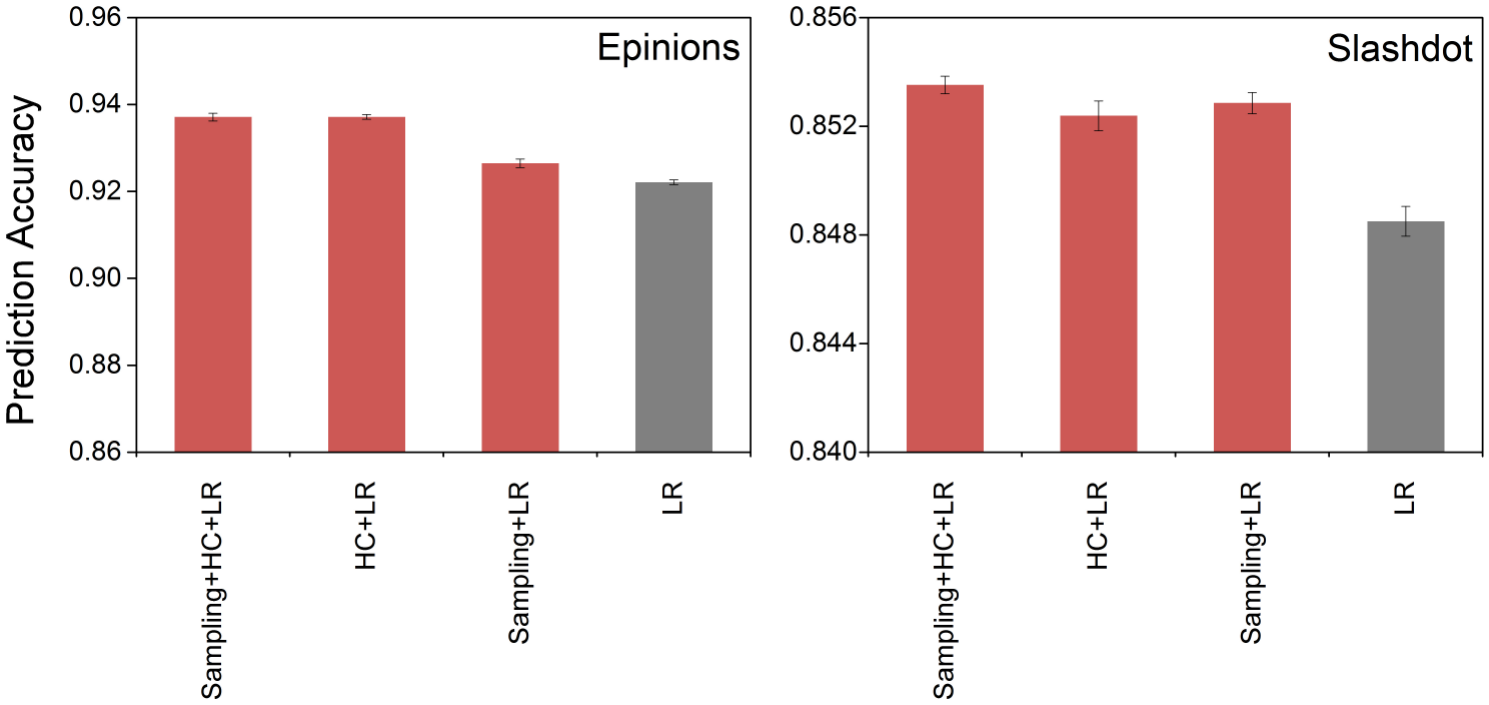
Prediction accuracy vs. strategies applied to Leskovec’s algorithm (LR). Prediction accuracy of Leskovec’s algorithm (LR) with K-means sampling (Sampling) and segment-based training framework (HC) are shown from left to right: applying K-means sampling (*K* = 400, sampling size = 3000) and HC to LR, applying HC to LR, applying K-means sampling (*K* = 400, sampling size = 3000) to LR, original LR. Experiments are conducted on a PC with Intel Core i3-3220 CPU @ 3.30 GHz, 8 GB of RAM and 1.5 TB of hard disk.

From Fig 4, we can see that prediction models like LR, Adaboost and ESS outperform the heuristic algorithms. It indicates that models concerning both structural balance theory and social status theory will work better. Also we can see “Balance” works better here than “Status”, however, it does not mean that the variables related to social status theory are not important. As discussed in the Preliminary section, social status theory can predict positive and negative relationships on the edges not embedded in any triadic structures (i.e. *EM* = 0), while structural balance theory can only handle predictions with triadic relationships. As the edges not embedded in any triadic relationships in Epinions and Slashdot, respectively, occupy 19.8% and 47.9% of all edges, those two theories complement each other here. Comparing with other algorithms, ESS considers both theories and establishes a more precise prediction model using segment-based training framework for SVM based on a much smaller and more representative training set. Furthermore, we employed the proposed strategies (K-means sampling and segment-based training framework) on Leskovec’s model. We can see from Fig 5 that both K-means sampling and segment-based training framework proposed in our paper can bring about higher prediction accuracy.

### Sensitivity analysis

**Fig 6:**
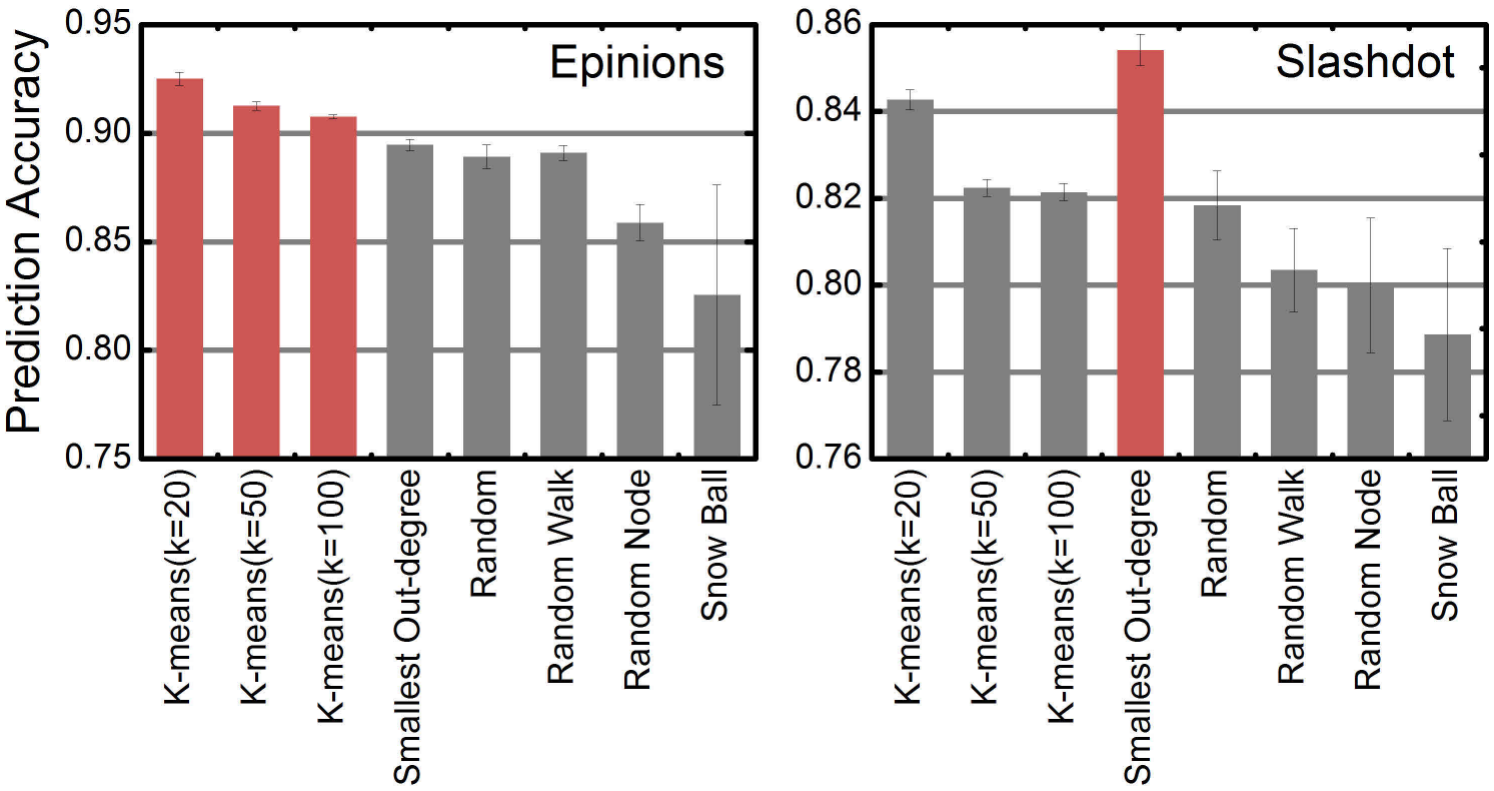
Prediction accuracy w.r.t. sampling strategies. Prediction accuracy based on different sampling strategies (k indicates the number of clusters). The size of compact training set is 100.

We compared the aforementioned three sampling strategies with other efficient and simple strategies [40] such as random node, snowball and random walk. Experiments were conducted on a PC with Intel Core i3-3220 CPU @ 3.30 GHz, 8 GB of RAM and 1.5 TB of hard disk. We limited the sampling size to 100, a relatively small number compared to the sizes of our datasets. As Fig 6 shows, SVMs trained on the datasets formed by K-means cluster strategy and smallest out-degree can achieve the highest prediction accuracy for Epinions and Slashdot separately. As for the other commonly used strategies which are simply based on the topology of the network, the prediction accuracy is relatively low. It indicates that, K-means sampling strategy is able to divide raw trainings set into several diverging clusters and pick representative instances from different clusters.

**Fig 7:**
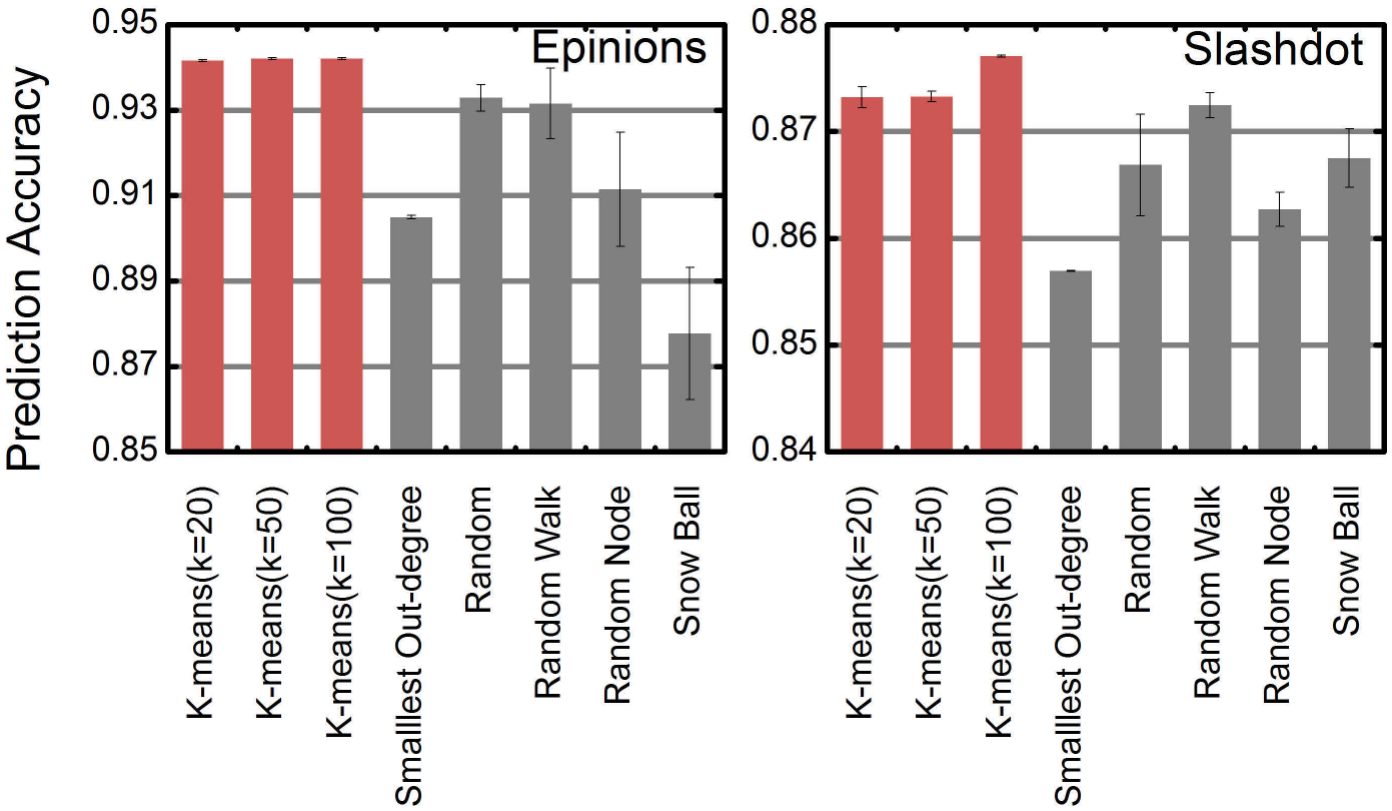
Prediction accuracy w.r.t. sampling strategies. Prediction accuracy based on different sampling strategies. The size of compact training set is 3000.

We also compared the sampling strategies when the sampling size is 3000. Fig 7 shows that using K-means cluster can achieve higher prediction accuracy than using others. As K-means can gather edges with similar topological structures and status of end points in each same cluster, it might be helpful for sampling representative edges from each cluster. Suppose the raw original training set contains *m* instances, the time complexity of K-means cluster is *O*(*Km*), which is much smaller than *O*(*m* ^3^) for training SVM. Thus, employing K-means cluster as our sampling strategy is effective and worthwhile.

**Fig 8:**
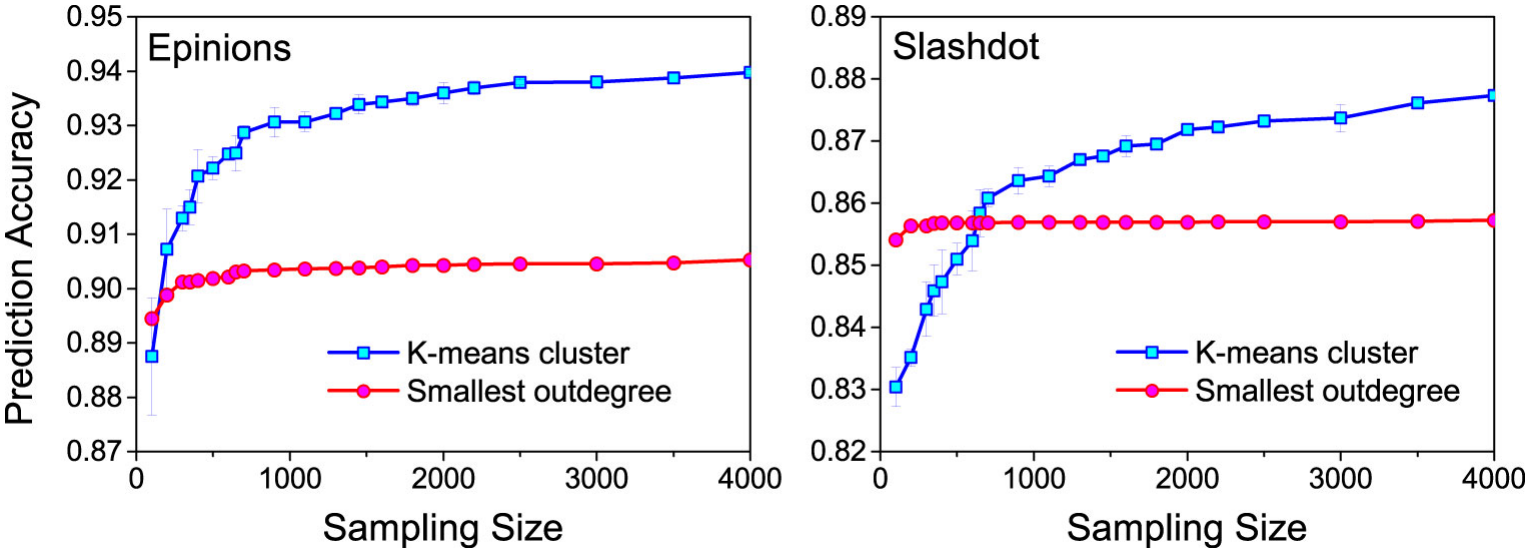
Prediction accuracy w.r.t. sampling size. The sampling size is from 20 to 4000. We set K = 100 for K-means cluster.

As Figs 6 and 7 present, the sampling size brings influence to prediction accuracy. Therefore, we moved further by studying how the sampling size effects prediction accuracy. Especially, we conducted our investigations based on two competitive strategies: K-means cluster and smallest out-degree. Fig 8 shows that the bigger sampling size becomes, the higher prediction accuracy K-means cluster achieves. However, prediction accuracy achieved by smallest out-degree remains monotonous with sampling size. For Epinions, using K-means cluster achieves higher prediction accuracy than using smallest out-degree. For Slashdot, prediction accuracy of using K-means cluster is lower than that of using smallest out-degree when the sampling size is below 400, but it grows rapidly from 100 to 1000. It indicates that the training set will contain more representative instances as its size grows. To achieve high prediction accuracy with low computational consumption, we decided to limit the sampling size to 3000 according to our experiment results.

We further investigated the influence of *K* (the number of clusters) of K-means sampling on the prediction accuracy. However, the influence from *K* is smaller than that from sampling size. That is, despite different *K*, K-means sampling strategy can always pick representative instances from different clusters. The stationary values of prediction accuracy obtained by using K-means cluster for different *K* are reported in Figure E in S1 File. For the Epinions dataset, the optimal *K* is 200; while for the Slashdot dataset, the optimal *K* is 400.

Additionally, ESS would still obtain the highest prediction accuracy when the training data were more limited (Figure F in S1 File). The representative training data sampled by K-means strategy may contribute to keep prediction accuracy of ESS steady. However, prediction accuracy of Adaboost remains more stable than LR when the training set is more limited, indicating that the strong classifier based on weak heuristic learners is not sensitive to the change of train/test ratio.

## Conclusions

Such positive and negative relationships like support and opposition, trust and suspicion are pervasive in social networks. Research on those can help us understand the propagation of relationships and comprehend the structures of networks. In reality, however, we cannot obtain the complete relationships of large scale networks. With the increasing scale of the social network, it is essential to design a highly efficient and accurate algorithm to predict those latent signs of relationships. We propose a novel prediction algorithm called ESS based on the theories of trust relationship in social networks. In order to reduce the computational resource consumption, we introduce efficient and effective strategies to select training instances from massive data, especially for K-means driven data sampling, which allows maintaining the representative link structure and information of the relationships in large-scale network, and thereby the prediction performance is promised. In the training phase, we use SVM and cross-validation strategy to ensure good generalization performance. We found that the classifier becomes more effective when *EM* increases because more information about relationships becomes available. In order to ensure high predictive accuracy for edges of low *EM*, we construct a segment-based training framework. In particular, we have tested our algorithm on two large-scale data sets: one from a consumer review site and the other from a technology-related news website.

In our experiments, we used AdaBoost to integrate existing algorithms into a highly accurate prediction rule, and compared it with our algorithm. The results show that our algorithm achieves the highest prediction accuracy based on sampled, compact training sets. The algorithms based on both complementary theories (i.e. structural balance theory and social status theory) will work better than algorithms concerning one single theory. A detailed analysis of the performance sensitivity of sampling size among different sampling strategies suggests that K-means cluster strategy outperforms others as the sampling size grows marginally, enabling us to construct a compact training set of a limited size. ESS works well and it is insensitive to different splits for training and testing, and different *K* of K-means cluster. Owning to the relatively low computational cost and high prediction accuracy, ESS is better suited for computing in large-scale networks.

## Supporting Information

S1 Datasets The signed networks investigated in this study. In this file, every directed edge is related to a separate row which contains the IDs of the two end nodes of the edge and an additional value which is provided as the sign of relationship.
(ZIP)

S1 File Supporting text and figures. 1. Entropy and structural balance theory (Figure A). 2. Hill climbing (Figure B). 3. Necessity of Hill Climbing (Figure C, Table A). 4. Necessity of sampling (Figure D). 5. Sensitivity analysis (Figures E and F).
(DOC)
