# Supplementary material for: Predicting Positive and Negative Relationships in Large Social Networks
Source: PLoS One. 2015 Jun 15;10(6):e0129530. doi: 10.1371/journal.pone.0129530 (PMC4468140; doi:10.1371/journal.pone.0129530)
Supplement: S1 File — 1. Entropy and structural balance theory (Figure A). 2. Hill climbing (Figure B). 3. Necessity of Hill Climbing (Figure C, Table A). 4. Necessity of sampling (Figure D). 5. Sensitivity analysis (Figures E and F). (DOC) [file pone.0129530.s002.doc]

**Supporting Information**

# Entropy and structural balance theory

In order to measure the average amount of information about structural balance theory contained in given data set *S*, we use the quantity for each type of 16 triangles illustrated in Fig. 3 in the article. More speciﬁcally, if we denote $p_{i}^{t}$ as the frequency for which instance *i* contains triangles belonging to type *t*, we can introduce the standard Shannon information entropy:

$I=-\frac{1}{\left|S\right|}\sum_{i\in S} \sum_{t\in\mathbf{T}_{i}} p_{i}^{t}\ln(p_{i}^{t})$ (A)

which is maximal ($\sum_{i=1}^{16} \frac{1}{16}\ln(\frac{1}{16})=2.77$) when the frequencies are the same (maximum disorder) and zero if each instance only contains triangles of a single type. Fig. A shows that instances with high *embeddedness^1^* (*EM*) will contain more information about structural balance theory.

**
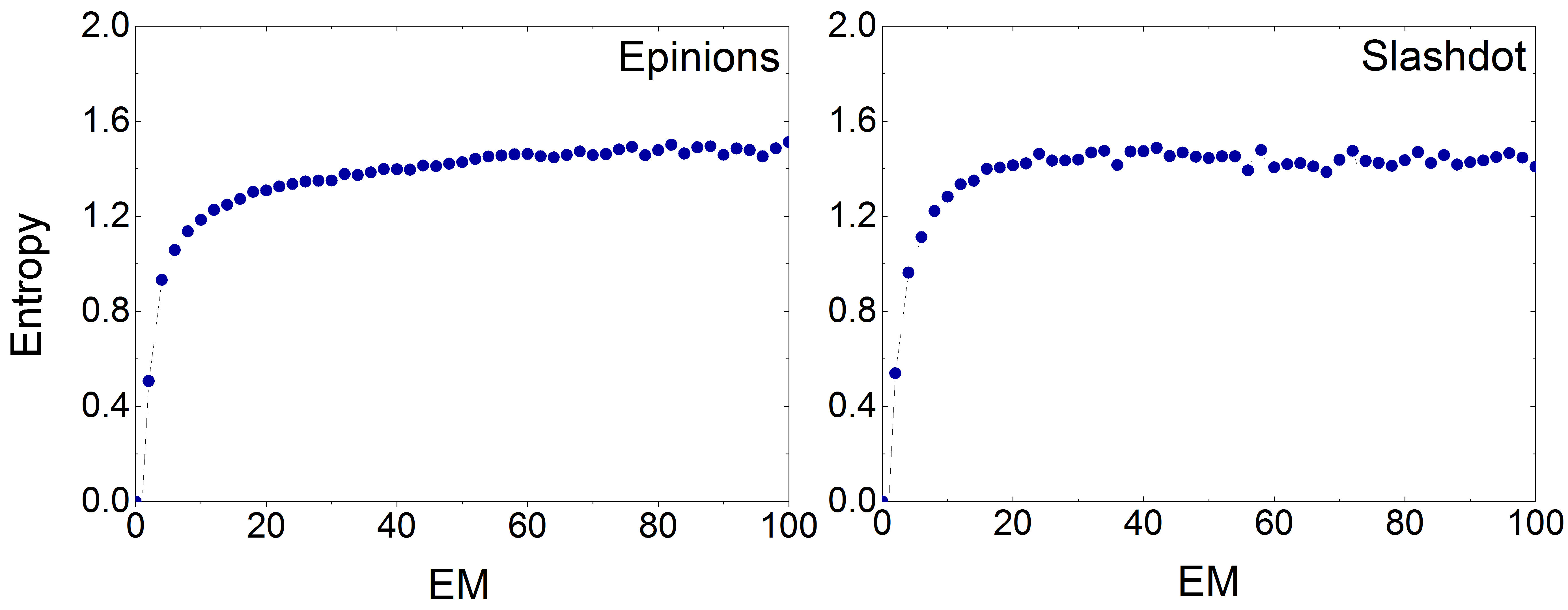
**

**Figure A. Information entropy of instances with different values of EM.**

# Hill climbing

A single classifier may achieve low prediction accuracy when classifying instances of low *EM*. It will be more appropriate to train sub-classifiers for instances of low *EM* and high *EM* respectively. The use of segment-based training framework will make our model more robust than using a single classifier whose predictive power is likely to vary a great deal among instances of different *EMs*. Here, we adopt Hill Climbing to find the best split of training instances. Basically, in a Hill Climbing heuristic, we start with an initial solution and generate more neighboring solutions. Then, pick the best and continue to generate neighboring solutions from it until there are no better solutions. In Hill Climbing, we need to know how to evaluate a solution, and how to generate "neighbors". Specifically, in this paper, we use overall cross-validation accuracy (Eq. B, same as Eq. 1 in the article) for evaluation, and move the current split point to generate "neighbors".

$p=\frac{|D_{1}|}{|D|}\cdot p_{C_{1}}+\frac{|D_{2}|}{|D|}\cdot p_{C_{2}}$ (B)

For each iteration Hill Climbing compares three classifiers (i.e. classifiers trained on three training sets that split in three ways) based on their overall cross-validation accuracy and chooses the split associated with the best one. Fig. B shows the pseudo-code describing Hill Climbing process used in our paper.

Here we give some interpretations of Eq. B, where $p_{C_{i}}$denotes the 10-fold cross-validation accuracy of sub-classifier trained on sub-training set *D_i_* :

$p_{C_{i}}=\frac{1}{10}\sum_{k=1}^{10} acc_{i,k}=\frac{1}{10}\sum_{k=1}^{10} \frac{1}{|V_{i,k}|}\sum_{s\in V_{i,k}} \delta(s_{l},\hat{s}_{l})$ (C)

where $acc_{i,k}$ denotes the prediction accuracy on validation data-set $V_{i,k}$ of *D_i_* in *k*^th^ fold, and $\delta(s_{l},\hat{s}_{l})$=1 if $s_{l}$(i.e. the label of sample *s*) equals $\hat{s}_{l}$(i.e. prediction of $s_{l}$) and 0 otherwise. Noting that validation data-sets of *D_i_* are of the same size under ideal conditions, Eq. B can be rewritten as:

$\begin{matrix} p=\frac{|D_{1}|}{|D|}\cdot p_{C_{1}}+\frac{|D_{2}|}{|D|}\cdot p_{C_{2}} \\ \text{=}\frac{|D_{1}|}{|D|}\cdot\frac{1}{10|V_{1,1}|}\sum_{k=1}^{10} \sum_{s\in V_{1,k}} \delta(s_{l},\hat{s}_{l})\text{+}\frac{|D_{2}|}{|D|}\cdot\frac{1}{10|V_{2,1}|}\sum_{k=1}^{10} \sum_{s\in V_{2,k}} \delta(s_{l},\hat{s}_{l}) \end{matrix}$ (D)

Now, making use of the fact that $\sum_{k=1}^{10} |V_{i,k}|=|D_{i}|$, we have

$\begin{matrix} p=\frac{1}{|D|}\cdot\left(\sum_{k=1}^{10} \sum_{s\in V_{1,k}} \delta(s_{l},\hat{s}_{l})\text{+}\sum_{k=1}^{10} \sum_{s\in V_{2,k}} \delta(s_{l},\hat{s}_{l})\right) \\ \text{=}\frac{\sum_{k=1}^{10} \sum_{s\in V_{1,k}} \delta(s_{l},\hat{s}_{l})\text{+}\sum_{k=1}^{10} \sum_{s\in V_{2,k}} \delta(s_{l},\hat{s}_{l})}{\sum_{k=1}^{10} |V_{1,k}|+|V_{2,k}|} \end{matrix}$ (E)

which gives us the average validation accuracy over the whole validation data including instances with either high or low *EM*.


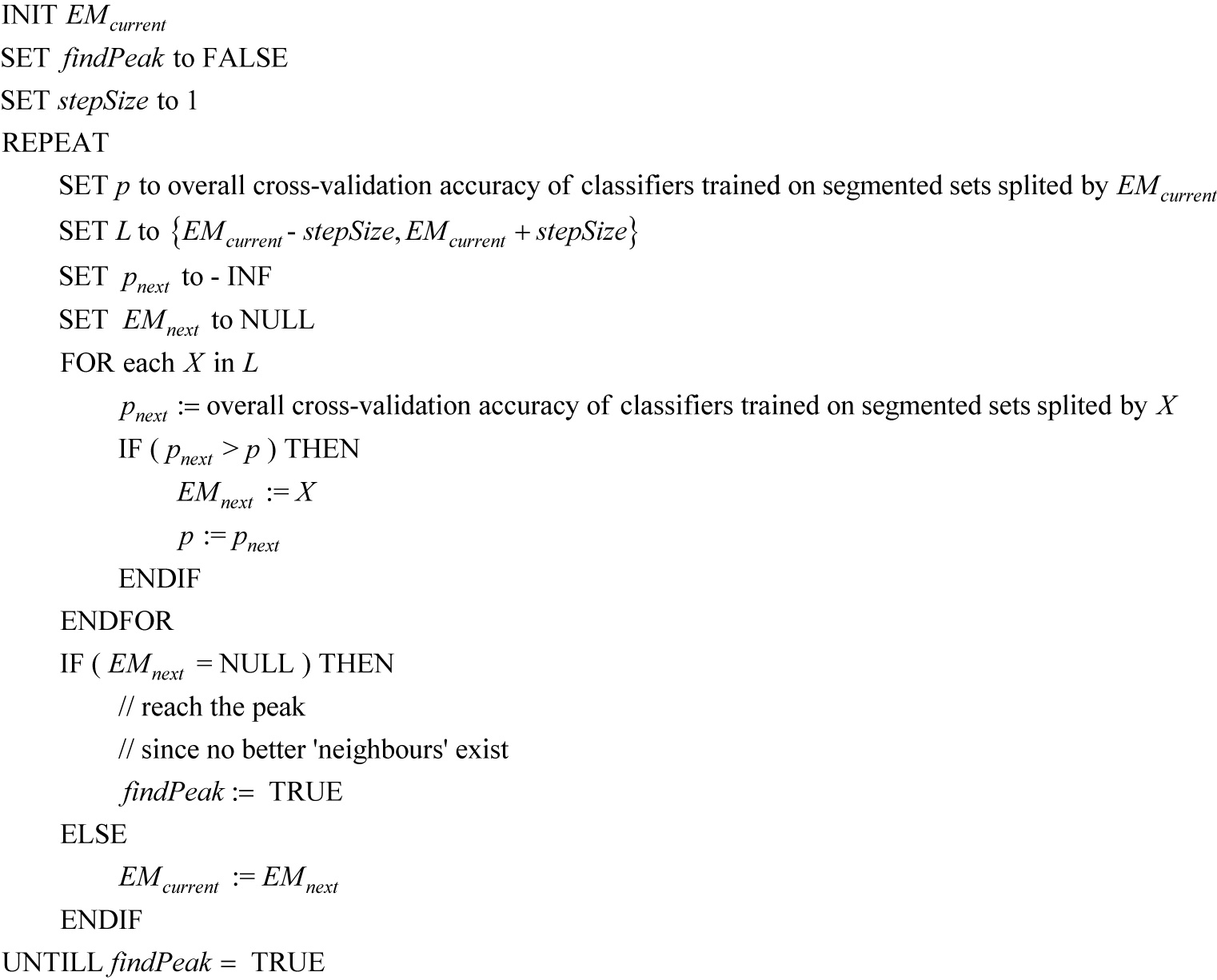


**Figure B. Pseudo-code of Hill-Climbing employed by our algorithm.**

# Necessity of Hill Climbing

Our algorithm ESS separates the training set and trains sub-SVMs on each sub-training set. In ESS, we adopt Hill Climbing to pick a segmentation point *d* of training set *D*, which splits *D* into sub-training sets *D*_1_ (where $0\leq EM<d$) and *D*_2_ (where $d\leq EM$). For each iteration Hill Climbing compares three classifiers (i.e. SVMs trained on three training sets that split in different ways) based on their overall cross-validation accuracy and chooses the split associated with the best one. After training, incoming instances will be classified by different sub-SVMs based on their values of *EM*.

We further investigate the necessity of Hill Climbing in this paper. Dependence of prediction accuracy on partition of training set is shown in Fig. C. It shows that sub-SVMs will achieve higher prediction accuracy than a single SVM (the red point). Also, there is a peak in the curve, which can be quickly found by Hill Climbing.


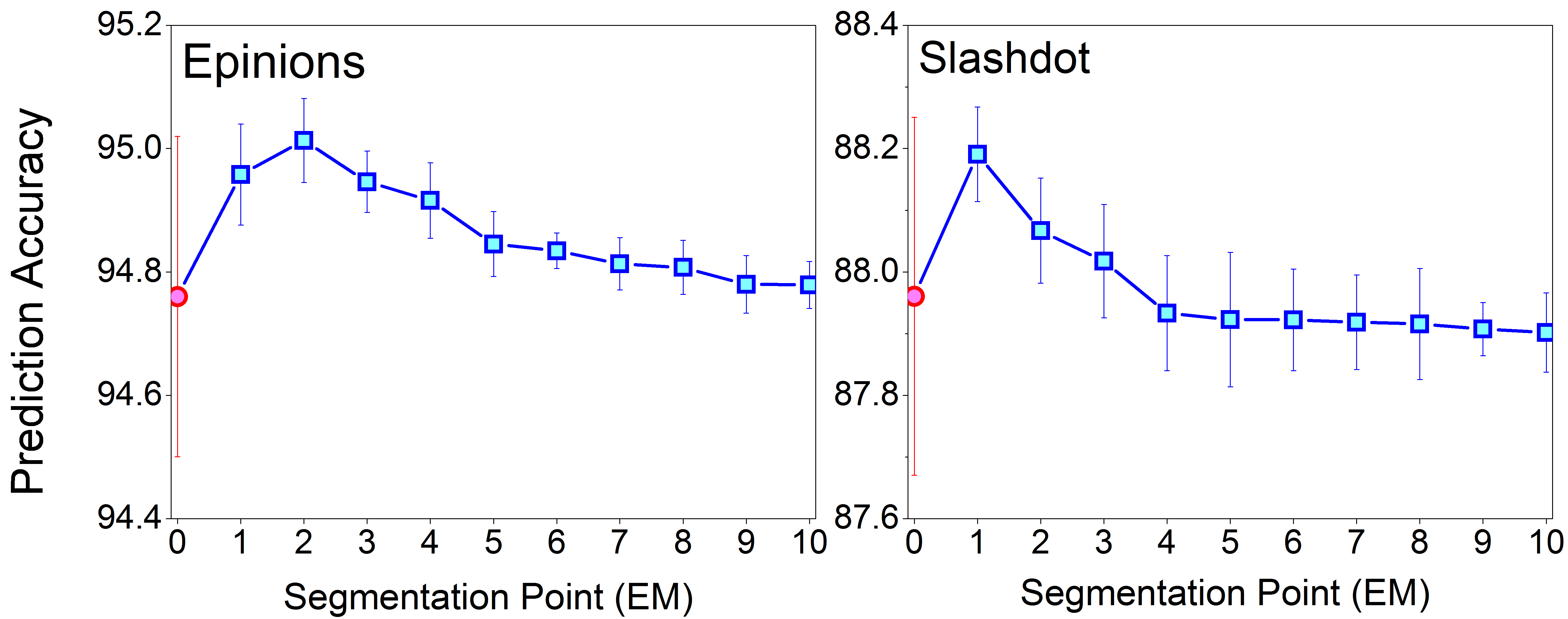


Figure C. Prediction accuracy w.r.t partition of training set. The blue points indicate prediction accuracy achieved by sub-classifiers (i.e. SVM) with different segmentation point. The entire training set consisting of 3000 instances is extracted by sampling strategy (K-means cluster, *K* = 400) from the raw training set. Train/test ratio is 9/1 as common. The optimal segmentation point is 2 for Epinions and 1 for Slashdot. Experiments are conducted on a PC with Intel Core i3-3220 CPU @ 3.30 GHz, 8 GB of RAM and 1.5 TB of hard disk.

We further scrutinized the sub-training sets, counted number of 16 types of triangles (see Fig. 3 in the article), and entropy of 16 types of triangles (see Eq. A). The statistic of the sub-training set are shown in Table A. As sub-training set 1 contains less information about structural balance theory than sub-training set 2, our algorithm ESS will focuses on status theory when training sub-SVM on sub-training set 1.

| **Table A | Statistic of the sub-training set** | | |
| --- | --- | --- |
| **#Slashdot** | **Sub-training set 1** | **Sub-training set 2** |
| **Range of *EM*** | *EM* < 2 | *EM* $\geq$ 2 |
| **Edges** | 297730 | 196551 |
| **Average number of 16 types of triangles** | 0.20 | 20.39 |
| **Entropy of 16 types of triangles** | 0 | 1.02 |
| **Ratio of negative attitudes to positive attitudes** | 0.32 | 0.27 |
| **#Epinions** | **Sub-training set 1** | **Sub-training set 2** |
| **Range of *EM*** | *EM* < 3 | *EM* $\geq$ 3 |
| **Edges** | 233073 | 524851 |
| **Average number of 16 triangles** | 0.54 | 68.63 |
| **Entropy of 16 triangles** | 0.09 | 1.28 |
| **Ratio of negative attitudes to positive attitudes** | 0.28 | 0.13 |

# Necessity of sampling

We tested the scalability and prediction accuracy of an SVM model (without sampling) on both datasets. As Fig. D shows, the running time grows rapidly with the size of training set, while the growth of prediction accuracy has slowed down. Those experiments were conducted on a PC with Intel Core i3-3220 CPU @ 3.30 GHz, 8 GB of RAM and 1.5 TB of hard disk.


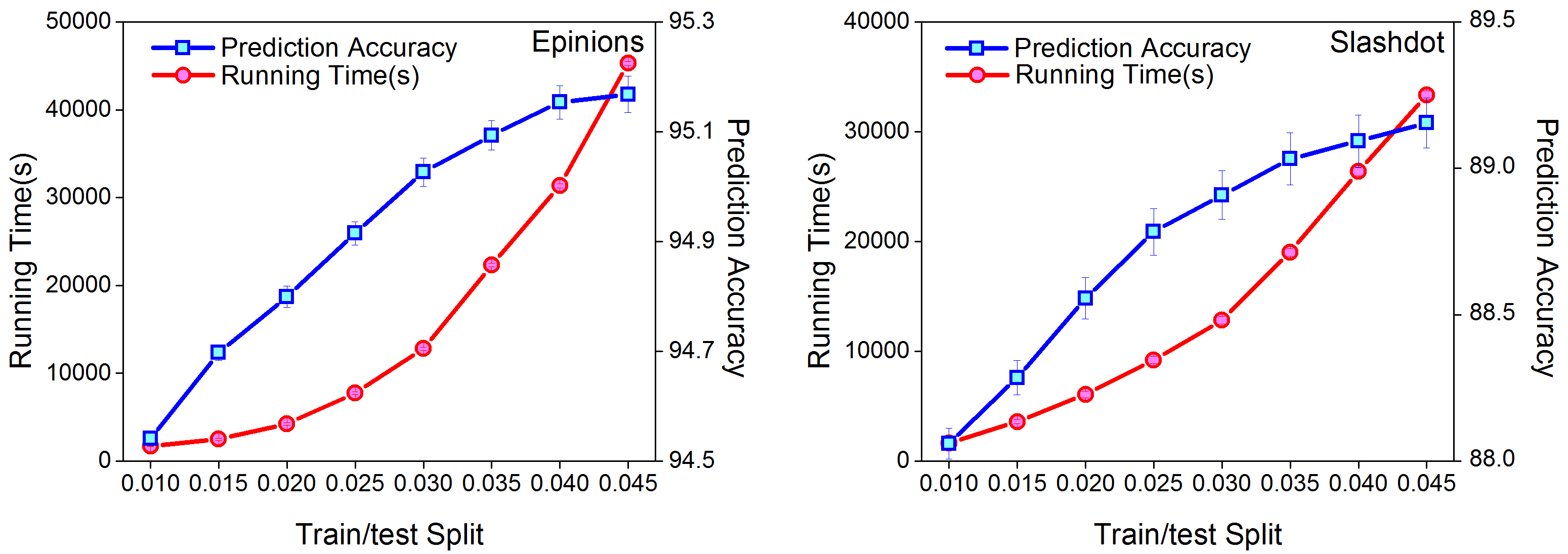


Figure D. Running time and prediction accuracy w.r.t train/test split.

However, our algorithm (using K-means sampling, *K* = 400, sampling size = 3000) only spends 253 seconds on Epinions and 848 seconds on Slashdot, with prediction accuracy higher than 95% and 88%, respectively. Using sub-sampling will reduce computation time and enable high prediction accuracy.

# Sensitivity analysis

We investigate the influence of *K* (the number of clusters) of K-means sampling on the prediction accuracy. However, the influence from *K* is smaller than that from sampling size. That is, despite different *K*, K-means sampling strategy can always pick representative instances from different clusters. The prediction accuracy obtained by using K-means for different *K* are reported in Fig. E. For the Epinions dataset, the optimal *K* is 200; while for the Slashdot dataset, the optimal *K* is 400.


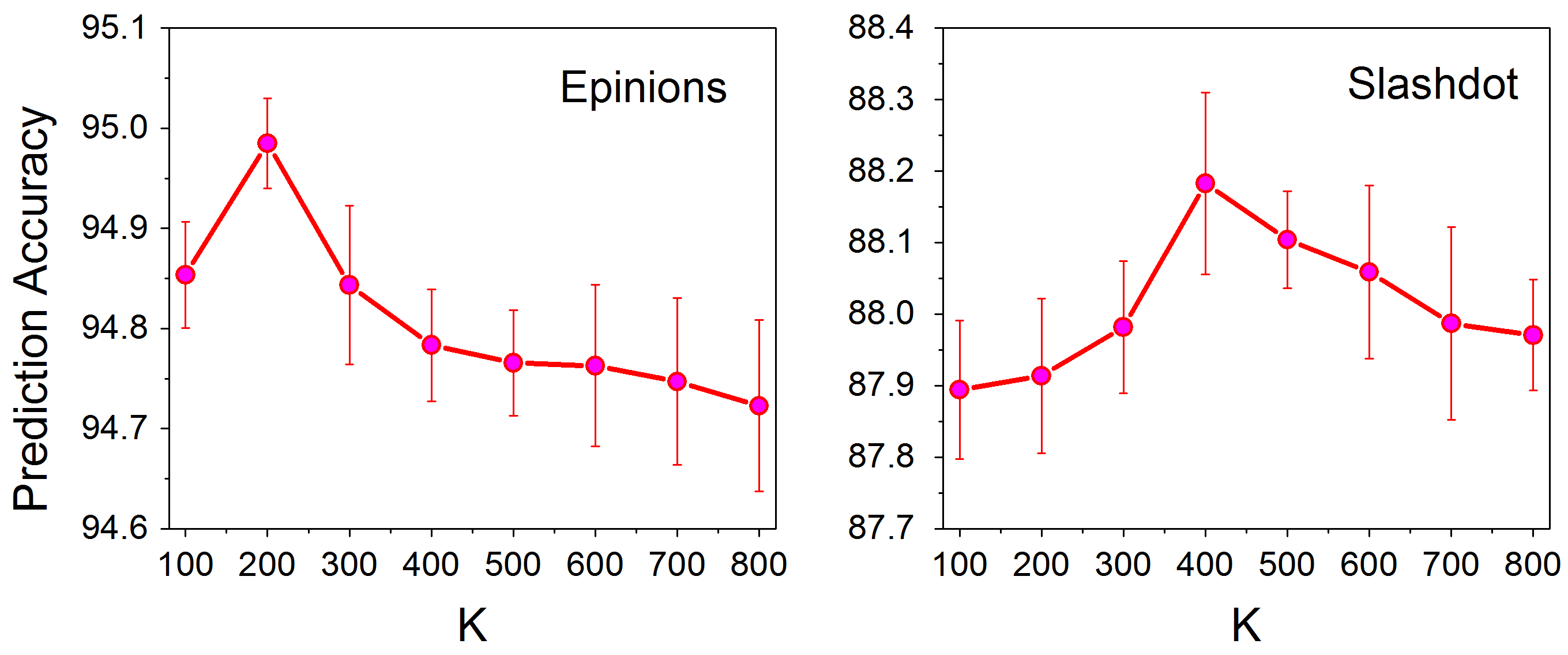


**Figure E. Prediction accuracy w.r.t. number of clusters.**

Additionally, ESS would still obtain the highest prediction accuracy with more limited size of training data (Fig. F). The representative training data sampled by K-means strategy may contribute to keeping prediction accuracy of ESS steady. However, prediction accuracy of Adaboost remains more stable than LR when the training set is more limited, indicating that the strong classifier based on weak learners might not be sensitive to the change of train/test ratio.


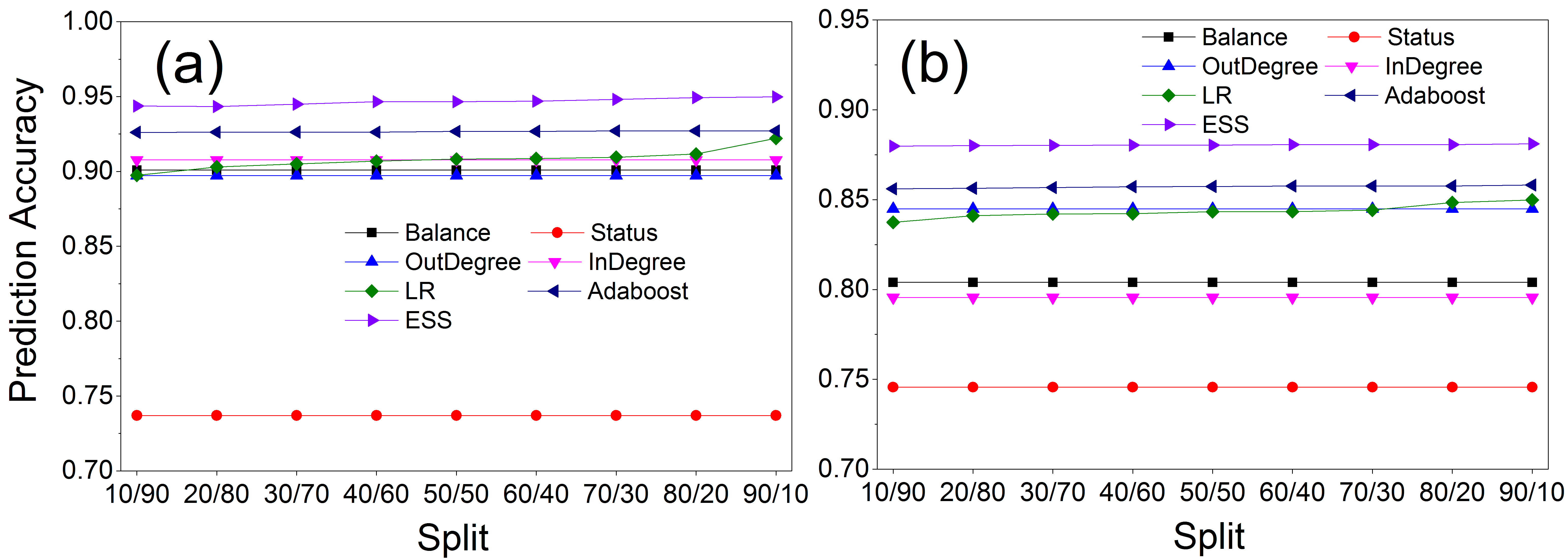


**Figure F. Accuracy w.r.t. split for training and testing.** Prediction accuracy of 7 algorithms with train/test ratio ranging from 10/90 to 90/10 in Epinions (a) and Slashdot (b). ESS is trained on a compact training set extracted by sampling strategy (K-means cluster, *K* = 400, sampling size = 3000) from the raw training set.

# Reference

[1] Easley, D., J. Kleinberg. *Networks, Crowds, and Markets: Reasoning About a Highly Connected World* (Cambridge University Press, Cambridge, 2010)

[2] Leskovec, J., Huttenlocher, D., Kleinberg, J. Predicting positive and negative links in online social networks. In: *WWW*, 641-650 (ACM, 2010).
